# A commentary on ‘A CT-based multitask deep learning model for predicting tumor stroma ratio and treatment outcomes in patients with colorectal cancer: a multicenter cohort study’

**DOI:** 10.1097/JS9.0000000000001431

**Published:** 2024-04-24

**Authors:** Wei Cheng, Lili Deng, Wen Liu

**Affiliations:** Department of Radiology, Jinshan Hospital, Fudan University, Shanghai, People’s Republic of China

The most prevalent gastrointestinal tract cancer worldwide and the second-leading cause of tumor-related mortality is colorectal cancer. In the past few decades, significant improvements have been made in the care of people with colorectal cancer. In recent years, advances in treatment strategies have played a significant role in raising survival rates. However, the overall survival of patients with advanced colorectal cancer remains poor. The choice of therapy options for people with colorectal cancer depends on TNM staging. Medical imaging is frequently used to preoperatively assess patient staging; nevertheless, its accuracy in determining TNM staging remains poor.

Amongst the stroma-related tumor microenvironment biomarkers, the tumor–stroma ratio (TSR), reflecting the interplay between tumor and stromal elements, has emerged as a stage-independent indicator for survival prognosis in patients with colorectal cancer. Yet, existing histological evaluation of TSR relies on post-surgery tissue samples, which introduces sampling bias due to intratumor spatial heterogeneity and limits accessibility. Moreover, visual estimation is prone to inconsistencies among pathologists, leading to discrepancies. Consequently, a noninvasive approach for assessing TSR status becomes imperative, enabling impartial and longitudinal tumor microenvironment evaluations.

Deep learning refers to the class of machine learning methods that make use of successively more abstract representations of the input data to perform a specific task^1^. These methods use training data to learn how these representations should be generated in a manner appropriate for the given task. By contrast, traditional machine learning uses handcrafted features to create representations of the input data that are applied to perform the task. In many applications, deep learning has been shown to be superior to other machine learning techniques and is expected to transform current medical practice. Convolutional neural networks have excelled in many image interpretation tasks and could be hypothesized to retrieve additional information from histopathology images.

Cui *et al*.^2^ performed a multicenter cohort study to develop a multitask deep learning (MDL) model to noninvasively predict TSR and prognosis in colorectal cancer. The results of the study indicated that MDL model based on preoperative CT (computed tomography) images effectively predicted TSR status and survival in colorectal cancer patients, offering valuable guidance for personalized treatment.

Regarding efforts, leveraging deep learning, which aims for tumor ‘stroma’ quantification based on whole-slide images (WSI), two studies have yielded encouraging results. Kather *et al*.^3^ trained a deep neural network to achieve tissue decomposition into tissue parts that could further be aggregated in a prognostic ‘deep stroma score’ for colorectal cancer. Their work supports the hypothesis that deep learning could be a significant aid in clinical prognostic settings. However, the clinical translation of their approach is still hampered by its semiautomatic nature (manual tumor region extraction from WSI was involved in their workflow), making it less objective and reproducible. It is noteworthy that they defined ‘stroma score’ as the weighted sum of various non-tumor tissues (desmoplastic stroma, lymphocytes, and adipose tissue). Hence, being unable to provide clear biological insights into the computational ‘black boxes’ is another weakness of their methodology. Geessink *et al*.^4^ semiautomated TSR assessment method using deep learning, the derived TSR could serve as an independent prognosticator for patients with rectal cancer. However, the study limitation was the need for human input for choosing user-provided hot spot. Further automation at the WSI level, beyond the limited hot-spot area, is still warranted.

The strengthening of this study is that it fills the gap by providing a fully automated method that requires no manual input on hematoxylin and eosin (HE)-stained WSI to facilitate an objective and fast TSR assessment. By reducing pathologists’ workload, this fully automatic workflow is well suited for its implementation in clinical practice. However, the use of adjuvant chemotherapy was not randomized, making it susceptible to selection biases. Prospective randomized clinical trials are necessary to validate the generalizability and clinical utility of this model.

## Ethical approval

None.

## Consent

None.

## Sources of funding

Shanghai Municipal Commission of Health (ZK2019B01).

## Author contribution

W.C.: wrote; L.D.: data analysis; W.L.: study design.

## Conflicts of interest disclosure

There are no conflicts of interest.

## Research registration unique identifying number (UIN)

None.

## Guarantor

Wei Cheng.

## Data availability statement

Not applicable

## Provenance and peer review

None.
